# A New Stalked Filter-Feeder from the Middle Cambrian Burgess Shale, British Columbia, Canada

**DOI:** 10.1371/journal.pone.0029233

**Published:** 2012-01-18

**Authors:** Lorna J. O'Brien, Jean-Bernard Caron

**Affiliations:** 1 Department of Ecology and Evolutionary Biology, University of Toronto, Toronto, Ontario, Canada; 2 Palaeobiology division, Department of Natural History, Royal Ontario Museum, Toronto, Ontario, Canada; Raymond M. Alf Museum of Paleontology, United States of America

## Abstract

Burgess Shale-type deposits provide invaluable insights into the early evolution of body plans and the ecological structure of Cambrian communities, but a number of species, continue to defy phylogenetic interpretations. Here we extend this list to include a new soft-bodied animal, *Siphusauctum gregarium* n. gen. and n. sp., from the Tulip Beds (Campsite Cliff Shale Member, Burgess Shale Formation) of Mount Stephen (Yoho National Park, British Columbia). With 1,133 specimens collected, *S. gregarium* is clearly the most abundant animal from this locality.

This stalked animal (reaching at least 20 cm in length), has a large ovoid calyx connected to a narrow bilayered stem and a small flattened or bulb-like holdfast. The calyx is enclosed by a flexible sheath with six small openings at the base, and a central terminal anus near the top encircled by indistinct openings. A prominent organ, represented by six radially symmetrical segments with comb-like elements, surrounds an internal body cavity with a large stomach, conical median gut and straight intestine. *Siphusauctum gregarium* was probably an active filter-feeder, with water passing through the calyx openings, capturing food particles with its comb-like elements. It often occurs in large assemblages on single bedding planes suggesting a gregarious lifestyle, with the animal living in high tier clusters. These were probably buried en masse more or less *in-situ* by rapid mud flow events.

*Siphusauctum gregarium* resembles *Dinomischus*, another Cambrian enigmatic stalked animal. Principal points of comparison include a long stem with a calyx containing a visceral mass and bract-like elements, and a similar lifestyle albeit occupying different tiering levels. The presence in both animals of a digestive tract with a potential stomach and anus suggest a grade of organization within bilaterians, but relationships with extant phyla are not straightforward. Thus, the broader affinities of *S. gregarium* remain largely unconstrained.

## Introduction

The Burgess Shale, discovered by Charles D. Walcott in 1909 between Wapta Mountain and Mount Field on Fossil Ridge (today's Walcott Quarry) in Yoho National Park, British Columbia, Canada, is famous for its exceptional preservation of soft-bodied animals dating from the Middle Cambrian period [1]. This site provides an incomparable window on the diversity and ecology of some of the first complex animal communities that thrived in the oceans directly in the aftermath of the Cambrian Explosion (e.g. [2], [3]).

In the last two decades the Burgess Shale fauna has received renewed interest most particularly thanks to the restudy of a number of Problematica [4], [5], [6]. Problematica, in the sense commonly applied to the Burgess Shale, are simply taxa which cannot be readily assigned to either modern phyla or similar high taxon-rank. These taxa (about twenty) were originally ‘shoe-horned’ within the phylogenetic brackets of extant phyla (e.g. [7]) and later interpreted as unknown body plans more or less equivalent to the level of phyla [8]. The former approach implies an earlier origin of body plans than compatible with the fossil record; the latter view effectively puts these taxa in a phylogenetic limbo and suggests a higher level of disparity in the Cambrian than observed today ([9] but see [3]). The reinterpretations of some Burgess Shale Problematica have been possible, at least in part, because of a) additional material collected in various Burgess Shale-type deposits, b) greater focus on potentially homologous characters and related to this, c) the widespread application of the stem and crown groups concepts [10]. In essence, many Burgess Shale fossils including supposed Problematica have been redefined as either early branches (stem-groups) of clades including either phyla or groups of phyla or even within crown-groups of phyla. Seen in this context, problematic taxa clearly play a central role in our understanding of how characters were both acquired and subsequently evolved within body plans. Much, however, remains controversial. Whether in the future a better understanding of problematic taxa can be achieved depends crucially on the discovery of new fossils and the recognition of convincing homologous characters. Nevertheless, it is clear that regardless of their correct affinities, problematic species also provide key ecological information on the structure and diversity of Cambrian communities. Here we describe a new stemmed animal, *Siphusauctum gregarium* n. gen. and n. sp., from the Burgess Shale (Tulip Beds locality), which despite its uncertain affinities extends the morphological and ecological range of benthic filter-feeders known in the Cambrian.

### Locality and stratigraphy

**Figure 1:**
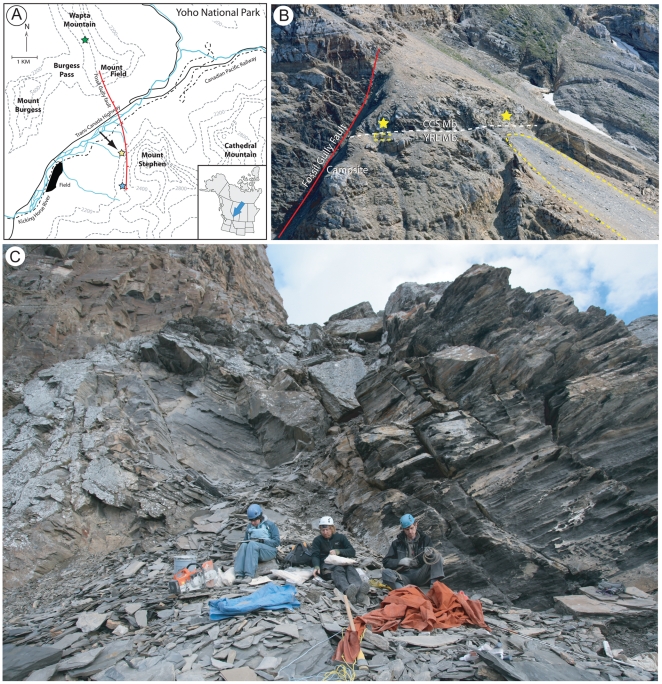
Geographical location of the Tulip Beds. A, position of the site (yellow star next to arrow), in relation to the Walcott Quarry (green star) and the Trilobite Beds (blue star). (The GPS coordinates for the locality are 51°24′06.1″ N, 116°27′06.4″W). B, telephoto of the Tulip Beds from Mount Field, showing the location of the main fossiliferous outcrops (yellow stars). Fossils were collected from two talus slopes (yellow dashed lines) or *in situ* in the Above Camp outcrop (yellow star on the left), now represented by a small quarry. The yellow star on the right indicates the approximate extent of the Above Camp fossil bearing layers to the southeast (distance between the two outcrops approximately 200 meters). The white dashed line indicates the boundary between the Campsite Cliff Shale Member (CCS) above and the Yoho River Limestone Member (YRL) below. C, close-up view of the Above Camp quarry in the summer of 2010 (yellow star on the left in B), showing the location of the fossiliferous beds excavated by the Royal Ontario Museum (small quarry just above crew members).

Much of our knowledge regarding the composition of the original Burgess Shale biota comes from the Walcott Quarry (on Fossil Ridge) (Fig. 1), which has yielded tens of thousands of specimens and close to two hundred species [2], [11]. Less well known are several other Burgess Shale-type deposits discovered in the vicinity of the Walcott Quarry by the Royal Ontario Museum (ROM) since the 1980's [12]–[14]. Among those, the Tulip Beds locality (formerly known as the S7 locality [14]), discovered in 1983 on Mount Stephen (Fig. 1) [13] is probably one of the most important new finds. This site is so named because of the presence of the distinctive “tulip-like” fossils, which are both abundant and unique to this locality (with perhaps one exception from Utah; see below). In addition to numerous algae, the Tulip Beds material contains a low diversity, but relatively abundant, faunal assemblage. This includes sponges, lobopodians, priapulid worms, arthropods and several enigmatic forms [14]. These are currently being investigated as part of a larger community study project.

Stratigraphically the site lies within the lower section of the Campsite Cliff Shale Member, five to ten meters above the top of the Yoho River Limestone Member [13] (Fig. 1). This is considered to fall within the lowermost *Bathyuriscus* zone [14], Series 3, Stage 5 of the Middle Cambrian. Fossil specimens were collected by several ROM field crews from *in situ* outcrop just south of Fossil Gully Fault (Above Camp S7) and two talus slopes. Fossils from talus slopes were collected below the Above Camp S7 outcrop (AC), and along a slope 200–300 m to the southwest which samples the same fossil bearing interval (known as the S7 slope).

The Campsite Cliff Shale Member comprises blocky, silty mudstones, which are interpreted to have been deposited in deeper and more distal conditions than those of the closely associated, and slightly older, Trilobite Beds [13]. The fossil bearing lithofacies are comprised of calcareous claystones, characterized by mm-scale event deposited muds. The position of the Campsite Cliff Shale member in relation to the Cathedral Escarpment is unclear but the nearest evidence of the Escarpment is located in an outcrop on the northeast shoulder of Mount Stephen, about 1,200 meters from the Tulip Beds. A detailed stratigraphic and geological study of the Tulip Beds and its relationship to the Cathedral Escarpment and other Burgess Shale localities is beyond the scope of this paper.

## Materials and Methods

**Figure 2:**
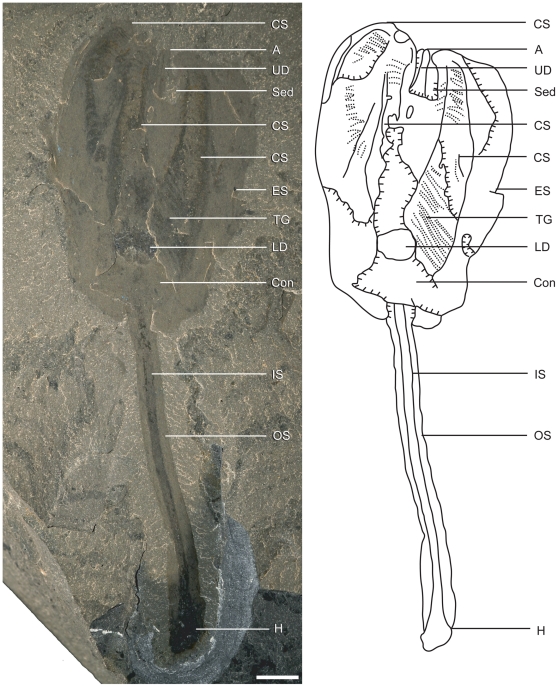
Paratype of *Siphusauctum gregarium* n. gen. and n. sp. (ROM 61413) and explanatory camera lucida drawing. Scale bar = 5 mm. Abbreviations: A - Anus, Con - Conical structure, CS - Comb Segments, ES - External Sheath, H - Holdfast, IS - Inner Stem, LD - Lower Digestive tract, OS - Outer Stem, Sed - Sediment, TG - Transverse Groove, UD - Upper Digestive tract.

**Figure 3:**
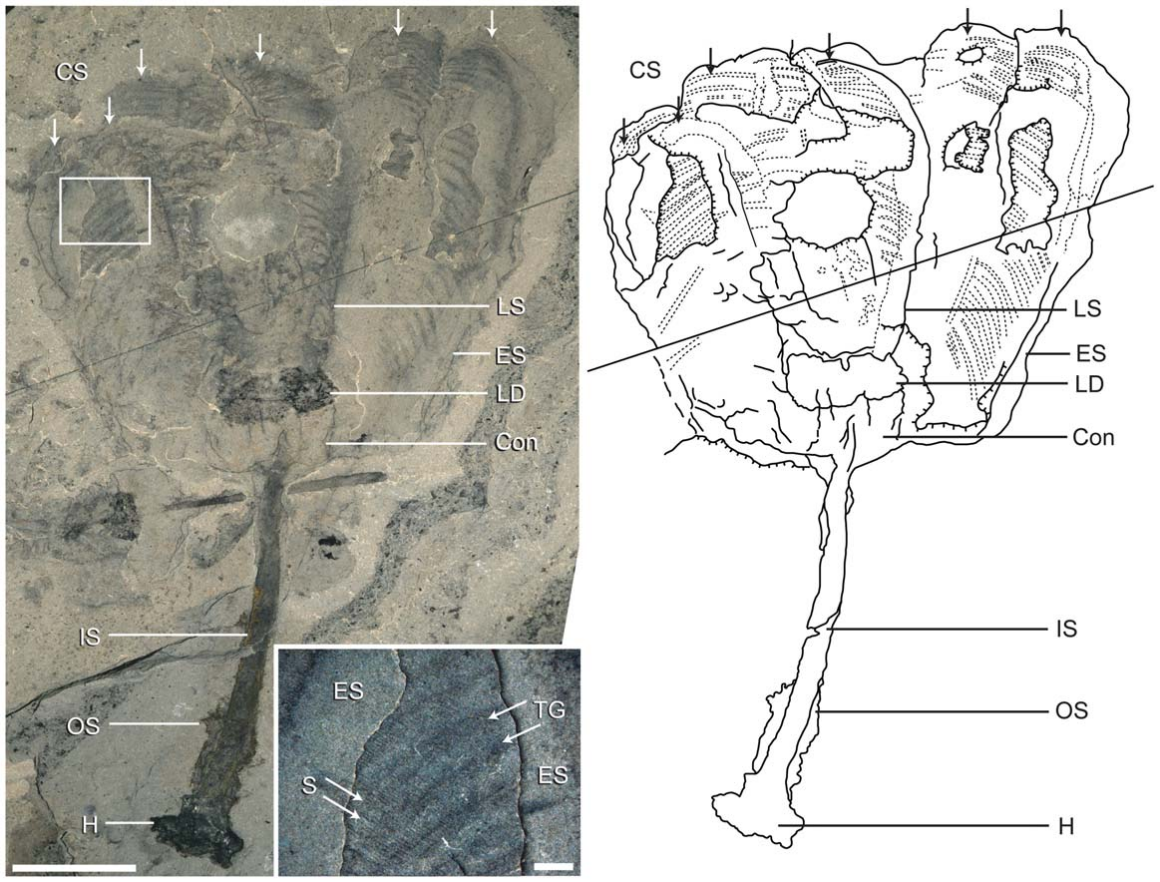
Holotype of *Siphusauctum gregarium* n. gen. and n. sp. (ROM 61414) and explanatory camera lucida drawing. Insert shows a larger view of the external sheath with the outer layer partially detached, showing fine striae and transverse individual grooves of the comb-like segment below. Scale bar = 10 mm, insert = 1 mm. Abbreviations: Con - Conical structure, CS - Comb Segments, ES - External Sheath, H - Holdfast, IS - Inner Stem, LD - Lower Digestive tract, LS - Longitudinal Suture line, OS - Outer Stem, S - Striae, TG - Transverse Groove.

**Figure 4:**
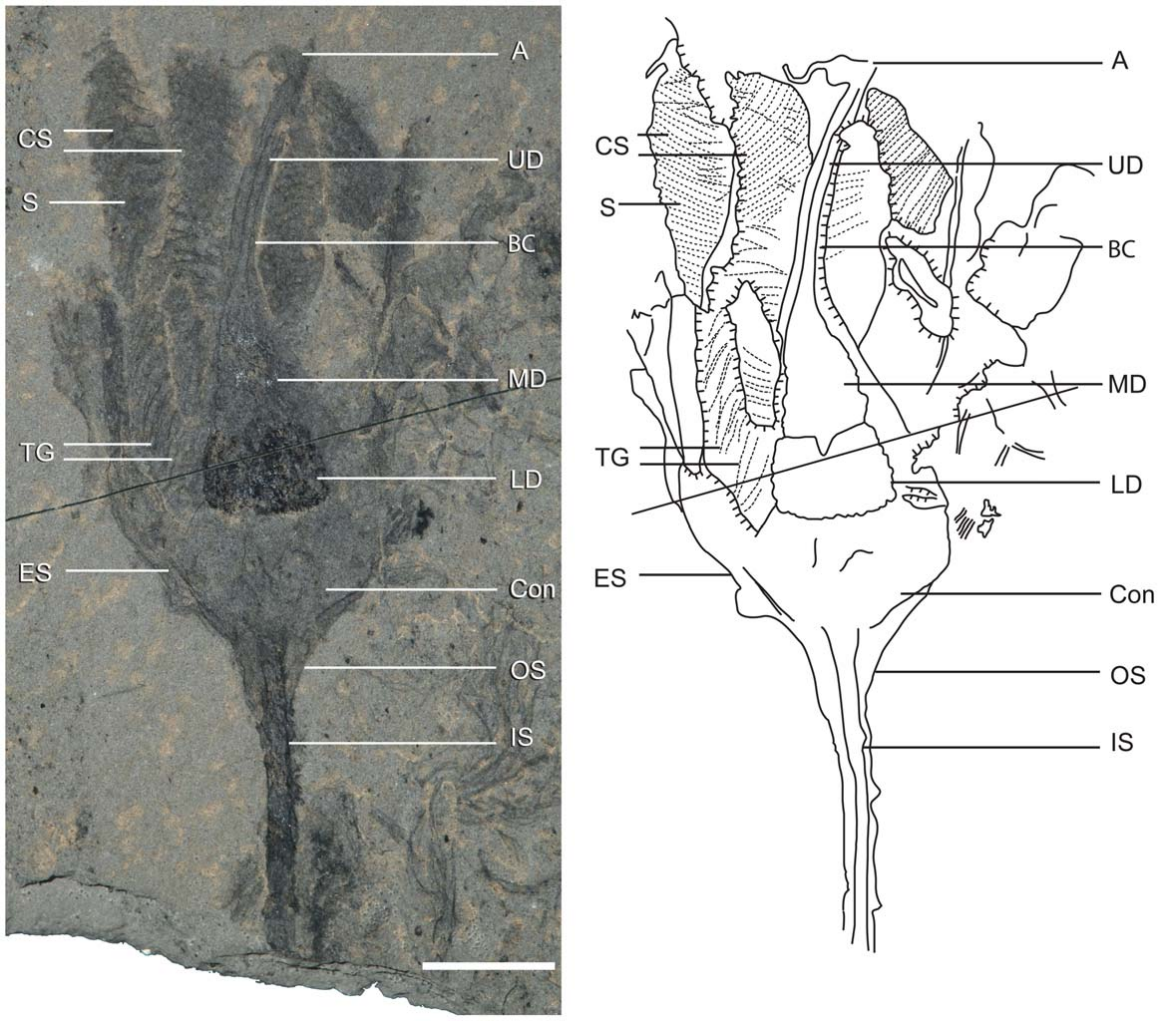
Paratype of *Siphusauctum gregarium* n. gen. and n. sp. (ROM 61415) and explanatory camera lucida drawing. Scale bar = 10 mm. Abbreviations: A - Anus, BC - Body Cavity, Con - Conical Structure, CS - Comb Segments, ES - External Sheath, IS - Inner Stem, LD - Lower Digestive tract, MD - Middle Digestive tract, OS - Outer Stem, S - Striae, TG - Transverse Groove, UD - Upper Digestive tract.

**Figure 5:**
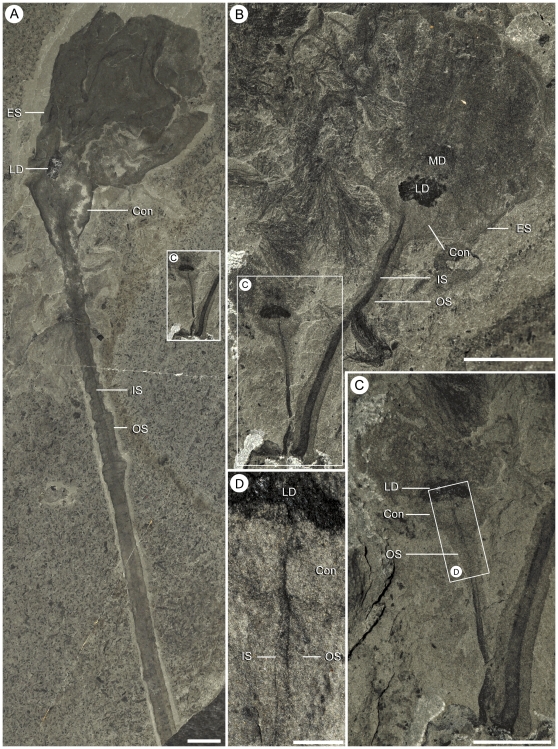
Range of sizes observed in *Siphusauctum gregarium* n. gen. and n. sp. A, the largest (ROM 61416) and one of the smallest (inset, see also B and C) specimens of *S. gregarium* to scale. B, Two specimens, side by side (ROM 61417). C, close-up of ROM 61417 (white frame in B, see also A). D, close-up of ROM 61417 (white frame in C) showing the connection of the inner stem to the base of the calyx. Scale bars: A, B = 10 mm, C = 5 mm, D = 1 mm. Abbreviations: Con - Conical structure, ES - External Sheath, IS - Inner Stem, LD - Lower Digestive tract, MD - Middle Digestive tract, OS - Outer Stem.

**Figure 6:**
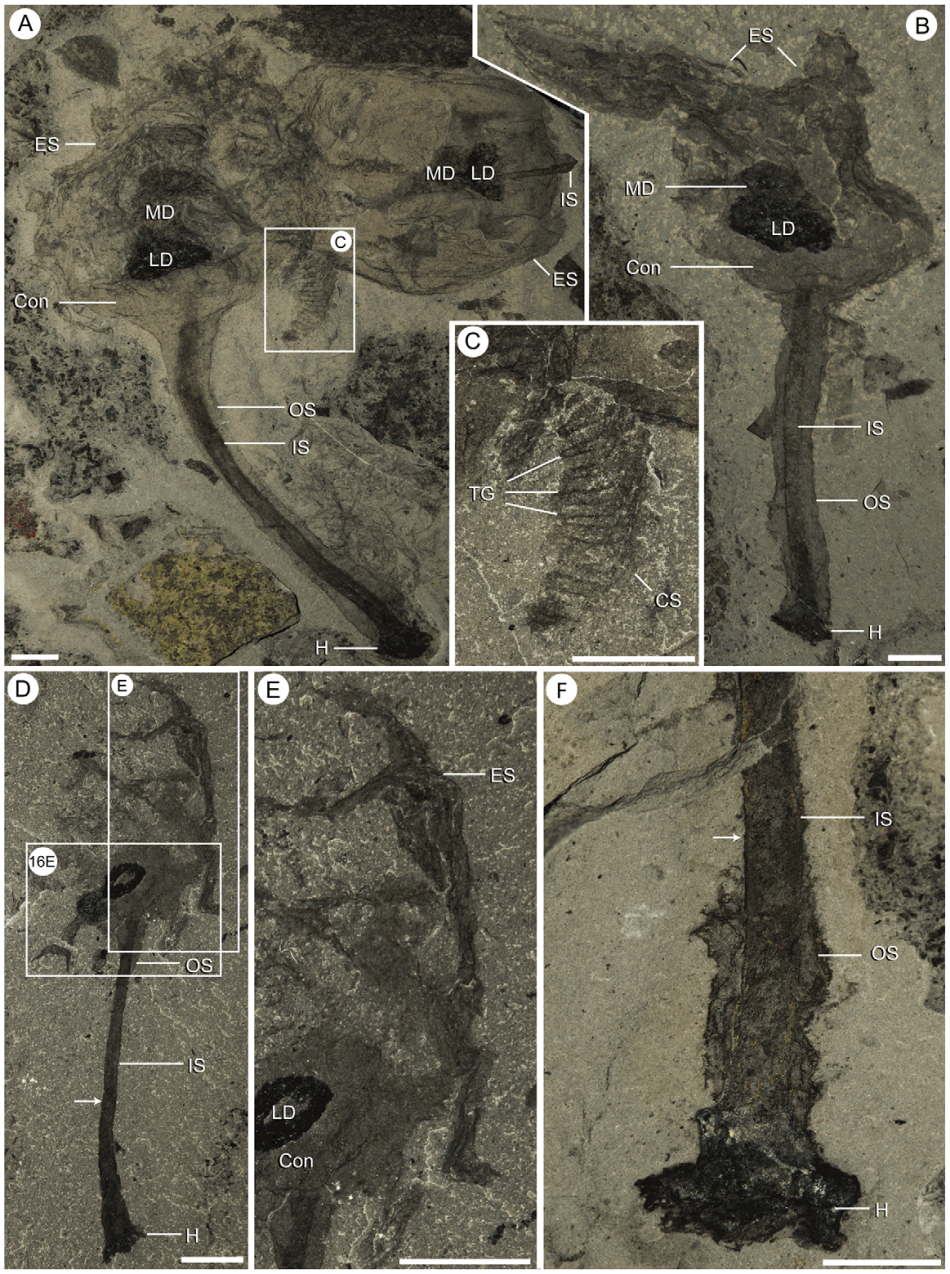
Taphonomic variation in *Siphusauctum gregarium* n. gen. and n. sp. A, complete calyx on the right and a decayed specimen on the left (ROM 61419). B, specimen with collapse and distortion of the upper part of the calyx (ROM 61420). C, Close-up of ROM 61419 (white frame in A), showing one disassociated comb-like segment. D, specimen where the outer stem has completely decayed (ROM 61418). E, Close-up of ROM 61418 (white frame in D), showing the remaining small pieces of the external sheath (other white frame, see Fig. 13E). F, partially decayed stem, showing the degradation of the outer stem (ROM 61414). Arrows indicate where the outer sheath has decayed. All scale bars = 5 mm. Abbreviations: Con - Conical structure, CS - Comb Segments, ES - External Sheath, H - Holdfast, IS - Inner Stem, LD - Lower Digestive tract, MD - Middle Digestive tract, OS - Outer Stem, TG - Transverse Groove.

**Figure 7:**
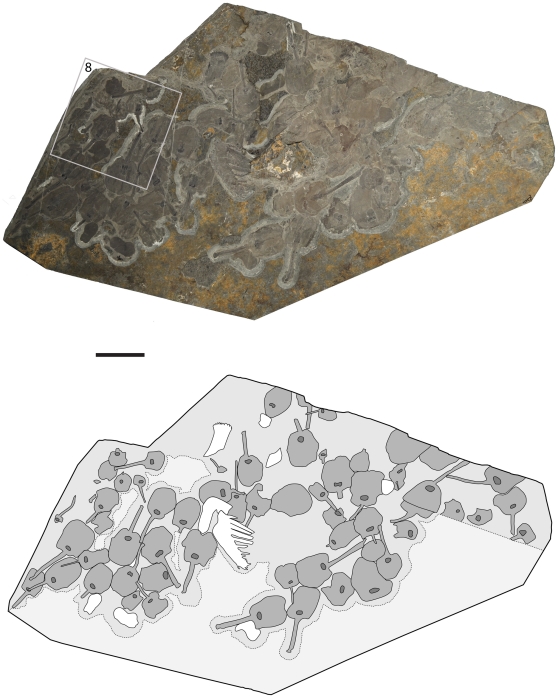
Cluster of 42 specimens of *Siphusauctum gregarium* n. gen. and n. sp. (ROM 61421). The line drawing shows the locations and orientations of the specimens (dark grey) on the slab. See Fig. 15, Table 1 and discussion on the orientations of the clusters. The grey box; see close-up in Fig. 8. Scale bar = 50 mm.

**Figure 8:**
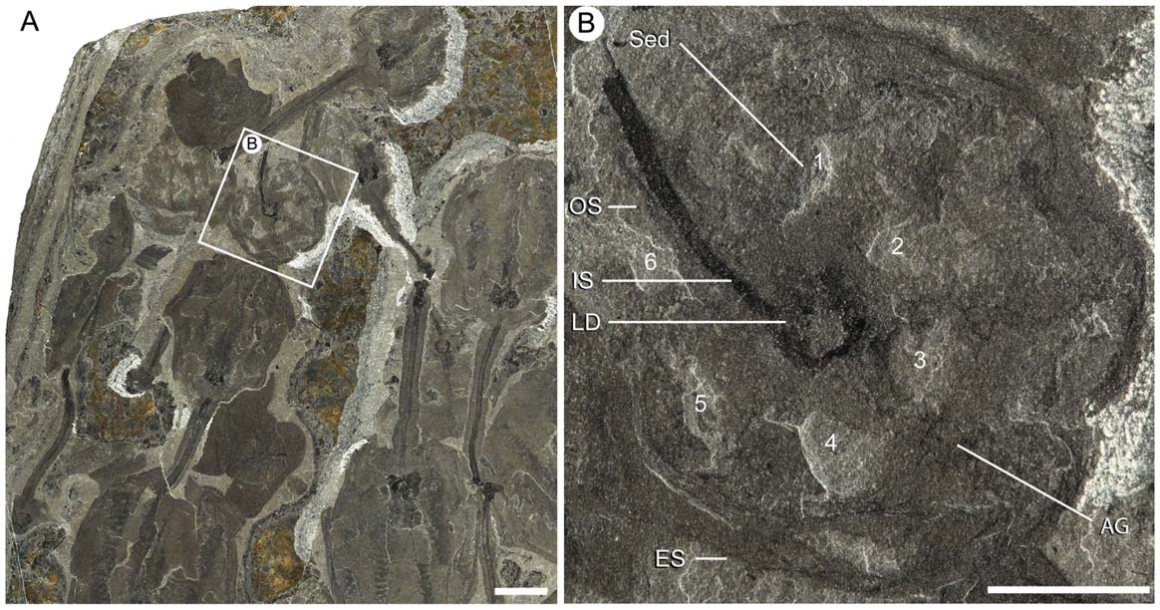
Specimen showing a ventral view of the calyx of *Siphusauctum gregarium* n. gen. and n. sp. (ROM 61421). A, Cluster of laterally preserved specimens with the single ventral view highlighted. B, close-up of a ventrally compressed specimen (white frame in A) showing the six axial grooves of the comb-like segments and six openings with sediment infill (numbered 1–6). Scale bars: A = 10 mm, B = 5 mm. Abbreviations: AG - Axial Groove, ES - External Sheath, IS - Inner Stem, LD - Lower Digestive tract, OS - Outer Stem, Sed - Sediment.

**Figure 9:**
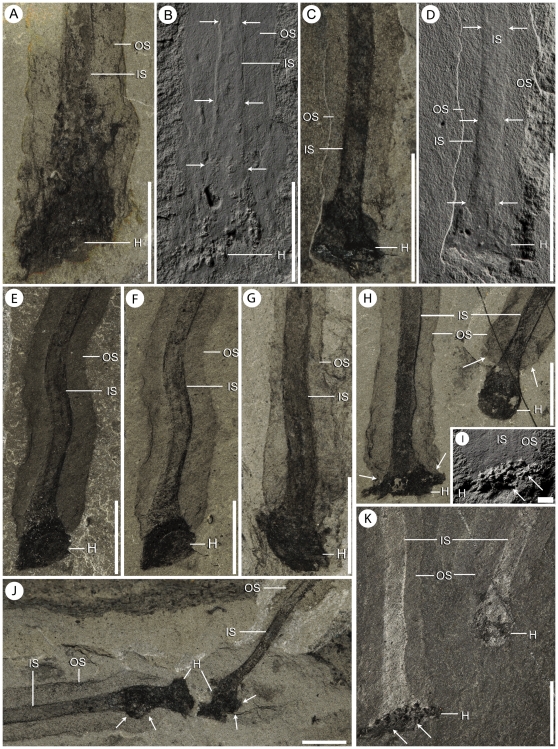
Shape and size variation within the stem and holdfast of *Siphusauctum gregarium* n. gen. and n. sp. A–B, stem and holdfast under polarized light (A) and covered in ammonium chloride (B), arrows indicate variation in thickness of the inner stem (ROM 61413). C–D, stem and holdfast under polarized light (C) and covered with ammonium chloride (D), arrows indicate change of thickness and relief in the inner stem (ROM 61421). E–F, specimen with bent stem, under normal lighting conditions (E) and under polarized light (F) (ROM 61422). G, specimen showing consistent thickness of the inner stem and terminating in a rounded holdfast (ROM 61419). H, K, two holdfasts with different morphologies, taken under polarized light (H) and under normal lighting conditions (K) (ROM 61423 – see complete specimens Fig. 10). Arrows in H indicate where the outer stem tapers in around the inner stem above the holdfast. I, a close-up of the left holdfast in K coated with ammonium chloride, the arrows indicate grainy texture. J, the terminal ends of two stems, arrows indicate areas of unusual swellings, possibly representing decay fluids (ROM 61421). Scale bars: A–H, J, K = 5 mm, I = 1 mm. Abbreviations: H - Holdfast, IS - Inner Stem, OS - Outer Stem.

**Figure 10:**
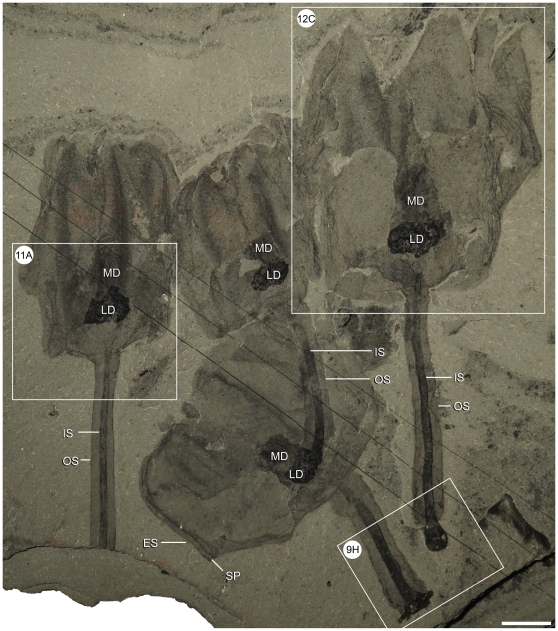
Overall shape and size variations within *Siphusauctum gregarium* n. gen. and n. sp. (ROM 61423). This small cluster shows variations in overall calyx shape and size, as well as, varying stem lengths and holdfast morphologies. Close-ups of the areas outlined by white frames refer to Figs. 9H, 11A and 12C. Scale bar = 10 mm. Abbreviations: H - Holdfast, IS - Inner Stem, LD - Lower Digestive tract, MD - Middle Digestive tract, OS - Outer Stem, SP - Sheath Protrusion.

**Figure 11:**
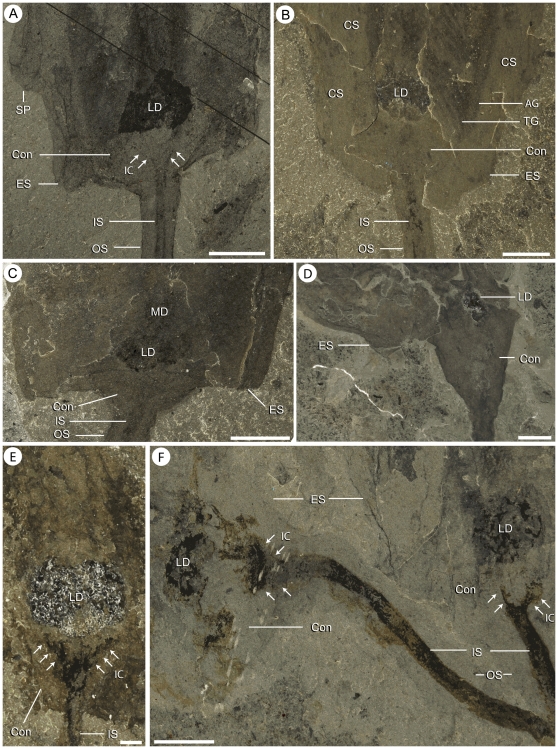
Shape variations in the lower calyx of *Siphusauctum gregarium* n. gen. and n. sp. A, flattened appearance, arrows indicate the internal conical structure (ROM 61423 – see complete specimen Fig. 10). B, rounded base (ROM 61413 – see complete specimen Fig. 3). C, flattened appearance (ROM 61422). D, conical appearance (ROM 61416 – close-up of the counterpart of Fig. 5A). E, base of a calyx with a distinctive widening inner conical structure (see arrows) (ROM 61425). F, two specimens showing variation within the inner conical structure (see arrows) (ROM 61424). Scales bars: A–D, F = 5 mm, E = 1 mm. Abbreviations: AG - Axial Groove, Con - Conical structure, CS - Comb Segments, ES - External Sheath, IC - Inner Conical structure, IS - Inner Stem, LD - Lower Digestive tract, MD - Middle Digestive tract, OS - Outer Stem, SP - Sheath protrusion, TG - Transverse Groove.

**Figure 12:**
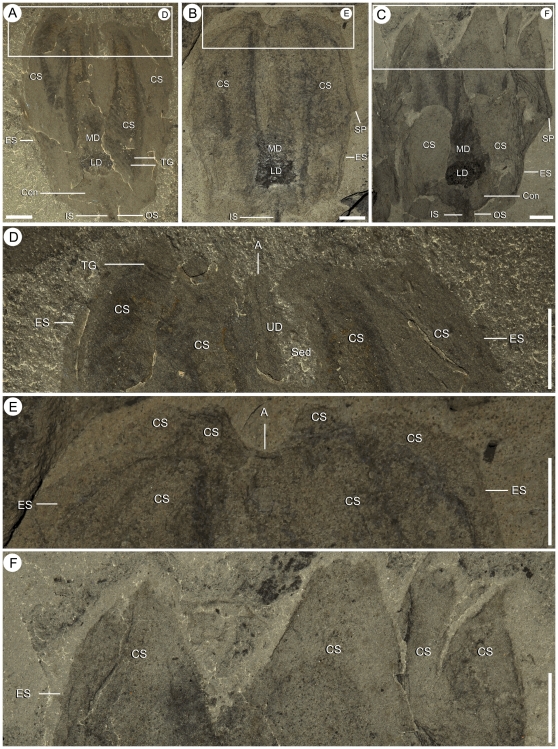
Shape variations in the upper calyx of *Siphusauctum gregarium* n. gen. and n. sp. A, D (close-up), rounded top (ROM 61413). B, E (close-up), corona-like appearance at the top (ROM 61426). C, F (close-up), calyx with extended comb-like segments (ROM 61423 – see complete specimen Fig. 10). Scale bars = 5 mm. Abbreviations: A - Anus, Con - Conical structure, CS - Comb Segments, ES - External Sheath, IS - Inner Stem, LD - Lower Digestive tract, MD - Middle Digestive tract, OS - Outer Stem, Sed - Sediment, SP - Sheath Protrusion, TG - Transverse Groove, UD - Upper Digestive tract.

**Figure 13:**
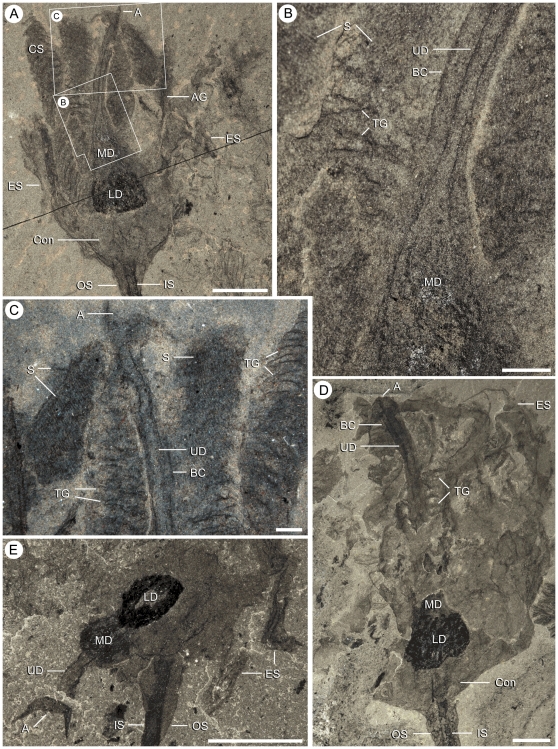
Internal anatomy of the calyx of *Siphusauctum gregarium* n. gen. and n. sp. A–C, paratype (ROM 61415) showing complete digestive tract (A) and close-ups of the gut (B and C) showing the gut enclosed within a body cavity, internal striae and transverse grooves . D, partially decayed specimen showing the overall shape of the digestive tract (ROM 61427). E, close-up of ROM 61418 (Fig. 6D), showing displacement of part of the digestive tract probably as a result of decay. Scale bars: A, D, E = 5 mm, B, C = 1 mm. Abbreviations: A - Anus, AG - Axial Groove, BC - Body Cavity, Con - Conical structure, CS - Comb Segments, ES - External Sheath, IS - Inner stem, LD - Lower Digestive tract, LS - Longitudinal Suture line, MD - Middle Digestive tract, OS - Outer Stem, S - Striae, TG - Transverse Groove, UD - Upper Digestive tract.

**Figure 14:**
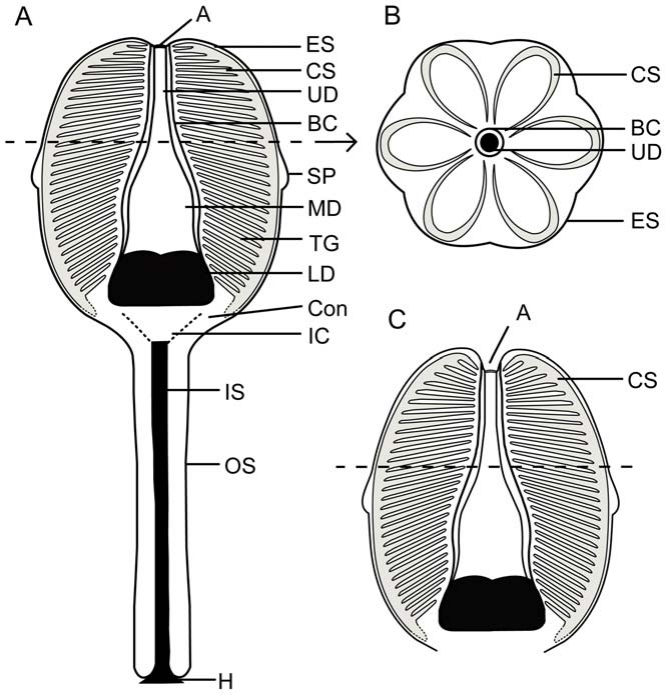
Schematics of the internal anatomy of *Siphusauctum gregarium* n. gen. and n. sp. A, longitudinal-section of the entire animal, with the major features labeled. B, cross-section of the calyx showing hexaradial symmetry. C, longitudinal-section of the calyx, showing raised comb segments above the level of the anus. Abbreviations: A - Anus, BC - Body Cavity, Con - Conical structure, CS - Comb Segments, ES - External Sheath, H - Holdfast, IC - Inner Conical structure with central tube, IS - Inner Stem, LD - Lower Digestive tract, MD - Middle Digestive tract, OS - Outer Stem, SP - Sheath Protrusion, TG - Transverse Groove, UD - Upper Digestive tract.

In total about 2,553 fossiliferous slabs (40% with part and counterpart) were collected from the Tulip Beds between 1983 and 2010, of which 70% come from the S7 slope outcrop (Fig. 1). *Siphusauctum gregarium* is one of the most abundant species from this locality, and our study is based on the examination of 1,133 specimens preserved on 281 slabs. The number of specimens per slab varies from one to more than 65. Sixty-eight percent of the specimens, including the largest clusters, come from the AC outcrop and its associated slope, of which 70% were collected *in-situ* within a 3 m thick interval, although exact stratigraphic information is unknown. The rest of the material (32%) comes from the southeast slope exposure. In total, about half of the specimens are known from both part and counterpart. Many specimens were prepared mechanically using an electric vibro-engraver with carbide bits to remove sediment coating specimen surfaces. The fossils were examined under polarized light with cross nicols (see [15]) and in some cases immersed in water to enhance details that are difficult to see under normal light conditions. Ammonium chloride was used to observe differences in relief of the specimens. Details of anatomy (Figs. 2, 3, 4, 5, 6, 7, 8, 9, 10, 11, 12, 13), size (Fig. 5) and the various modes of preservation (Fig. 6) were qualitatively and in some cases quantitatively recorded (see also Fig. 14 for anatomical schematic and Tables 1 and 2). Camera lucida drawings were made of the holotype and two paratypes. The lines with ticks on one side in the figures represent small escarpments resulting from breakage of different fossil layers; the orientation of the ticks indicates the lower level. Institutional abbreviations: MCZ – Museum of Comparative Zoology, Harvard, Massachusetts, USA. ROM – Royal Ontario Museum, Toronto, Canada. USNM – Smithsonian Institution, National Museum of Natural History, Washington, DC, USA. All necessary permits for the collection of fossils were obtained from Parks Canada. Collection and research permits were granted to D. Collins and J.-B. Caron between 1983 and 2010.

**Table 1:** Measurement parameters related to Fig. 15.

	Cluster A (ROM 61421)	Cluster B (ROM 61428)	Cluster C (ROM 61429)
**Number of Observations**	42	69	82
**Mean Vector (µ)**	40.7°	12.3°	21.3°
**Length of Mean Vector (r)**	0.32	0.09	0.18
**Concentration**	0.68	0.18	0.37
**Circular Variance**	0.68	0.91	0.82
**Circular Standard Deviation**	86.0°	126.1°	105.9°
**Rao**'**s Spacing Test (U)**	150	135	138
**Rao**'**s Spacing Test (p)**	0.10>p>0.05	0.50>p>0.10	0.50>p>0.10

Rao's Spacing test of significance, based on circular uniformity, indicates that these populations do not have an orientation significant from random in the direction of the specimens.

**Table 2:** Overall dimensions of *Siphusauctum gregarium* n. gen and n. sp.

	Mean	SD	Max	Min	N
**Stem L**	31.6	12.5	147.0	9.5	177
**Stem W**	4.7	1.3	11.2	1.4	200
**Stem inner W**	1.7	0.4	3.7	0.7	200
**Calyx L**	36.1	10.1	76.4	9.0	208
**Calyx W**	29.6	7.5	49.8	6.6	209
**Total L**	66.5	20.5	223.4	18.5	177
**Stomach W**	8.1	2.4	15.9	3.0	210
**Stomach L**	4.8	1.3	9.2	1.7	210
**Cone L**	5.5	2.1	20.2	2.6	210
**Holdfast W**	4.5	1.7	10.8	1.8	125
***Ratio***					
**Stem L/W**	7.0	2.3	21.3	3.0	177
**Stem W/Inner W**	2.7	0.6	5.8	1.5	200
**Calyx L/W**	1.2	0.2	1.9	0.7	207
**Total L/W**	2.3	0.5	4.7	1.3	176
**Stem L/Calyx W**	0.9	0.2	1.9	0.5	177
**Calyx W/Stem W**	6.5	1.4	14.2	3.4	200
**Calyx W/Stomach W**	3.8	0.7	8.3	2.0	209
**Stomach L/W**	1.7	0.4	3.4	0.7	210
**Calyx L/Cone L**	6.8	1.7	14.7	3.1	208

All measurements are in millimeters.

### Nomenclatural acts

The electronic version of this document does not represent a published work according to the International Code of Zoological Nomenclature (ICZN), and hence the nomenclatural acts contained in the electronic version are not available under that Code from the electronic edition. Therefore, a separate edition of this document was produced by a method that assures numerous identical and durable copies, and those copies were simultaneously obtainable (from the publication date noted on the first page of this article) for the purpose of providing a public and permanent scientific record, in accordance with Article 8.1 of the Code. The separate print-only edition is available on request from PLoS by sending a request to PLoS ONE, 185 Berry Street, Suite 3100, San Francisco, CA 94107, USA along with a check for $10 (to cover printing and postage) payable to “Public Library of Science”.

In addition, this published work and the nomenclatural acts it contains have been registered in ZooBank, the proposed online registration system for the ICZN. The ZooBank LSIDs (Life Science Identifiers) can be resolved and the associated information viewed through any standard web browser by appending the LSID to the prefix “http://zoobank.org/”. The LSID for this publication is: urn:lsid:zoobank.org:pub:94B075BE-B08D-4026-BF6F-F2D5383E1B54.

## Results

### Taphonomy

Evidence of soft-deformation suggests that this animal was entirely non-mineralized. Like other Burgess Shale fossils [16], *S. gregarium* is preserved as compressed carbonaceous and aluminosilicate films parallel to bedding in a dark mudstone. All specimens were buried laterally or obliquely along the longitudinal axis (e.g. Fig. 7), except for one vertically compressed individual showing the radial axis (Fig. 8). The holdfast is occasionally preserved with a granular appearance at its base, which might suggest that it was once attached to coarser sediments (Fig. 9B,I,K). However, it is also possible that these granular elements represent differential tissue reactivity and partial decay of the holdfasts accompanied by early diagenetic mineralization. Examples of early diagenetic mineralization of cavities or tissues in other Burgess Shale species (e.g. midgut glands of *Leanchoilia* [17], gut diverticulae of *Burgessia* [16], eyes in *Nectocaris* [5]), may not be directly comparable, but at least, they suggest that such type of differential preservation is possible.

**Figure 15:**
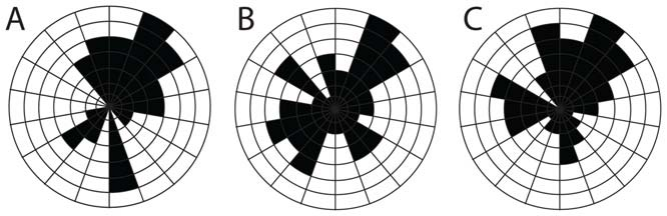
Rose diagrams showing the relative orientation of specimens of *Siphusauctum gregarium* n. gen and n. sp. on three different slabs. A, ROM 61421. B, ROM 61428. C, ROM 61429. The measurements are relative as the original *in-situ* orientation is unknown. (See Table 1).

**Figure 16:**
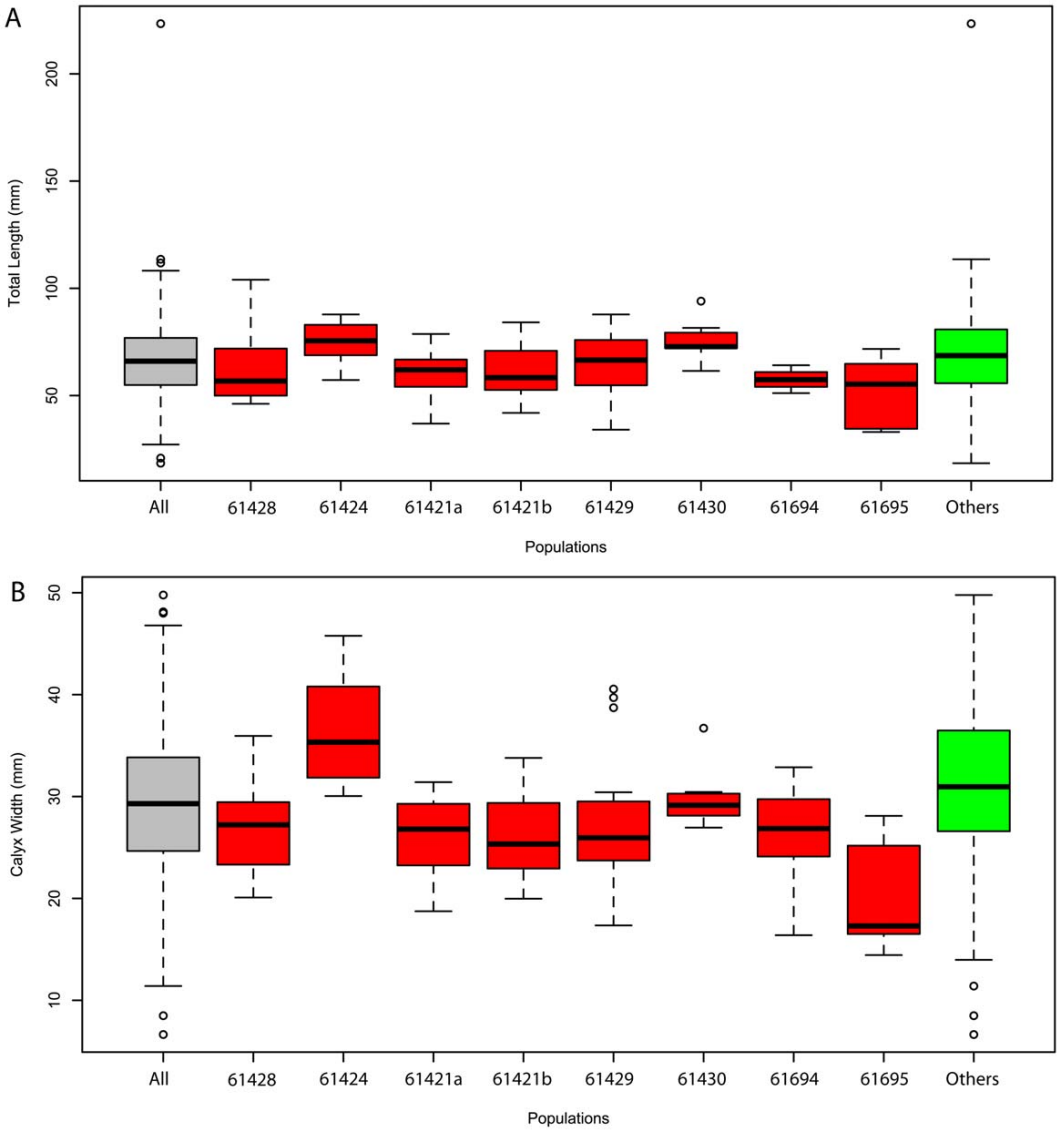
Population variations of *Siphusauctum gregarium* n. gen. and n. sp within particular slabs. Box and whisker plots: of total length (A) and calyx width (B). Grey indicates the entire data set, red indicates separate slabs with five or more individuals, and green indicates individual specimens and clusters of less than five specimens. The thick black lines indicate the mean value and the circles indicate outliers. Numbers below box and whisker plots refer to ROM numbers. N = 177.

The quality of preservation varies greatly. It ranges from specimens with barely visible outlines or at least with some evidence of decay and dissociation (Fig. 6), to complete specimens with internal details preserved (Figs. 7, 8). While some variations could be attributed to diagenetic processes including post-burial decay, biostratinomic factors can be demonstrated in a number of specimens. For instance, elements of the calyx are sometimes disarticulated and found in close proximity with other parts of the same individual, including the stem (Fig. 6A, C). Such associations suggest pre-burial decay and possibly at most minimal transport after decay had started and during burial. The lack of significant transport is also suggested by the random orientations of specimens in clusters (Table 1, Fig. 15) and the lack or limited amount of sediment between specimens (Fig. 7). The fact that specimens within clusters are about the same size (Fig. 16) and tend to have similar preservation reinforces the view that entire populations were rapidly smothered and were not significantly transported.

The external sheath decayed faster than the internal elements of the calyx. Only in specimens where decay is evident can the internal elements of the calyx (e.g. the feeding apparatus and the gut) be clearly seen (Figs. 3, 13). The loss of the external sheath can also result in internal elements appearing out of life position (e.g. comb-like segments splayed outwards, appearing twisted, or detached from the main body) (Figs. 6A, C, D, 13E).

The inner layer of the stem, the conical structure and the gut are the most decay-resistant parts of the animal. The stems are generally preserved straight or slightly bent (e.g. Figs. 5, 6A, 7, 9), have a uniform width, and have never been observed to be detached from the calyx. Like the external sheath of the calyx, the outer layer of the stem evidently decayed more rapidly so that in the most decayed specimens only the inner layer of the stems is preserved. The inner layer of the stem has noticeably higher relief than the outer layer (Fig. 9B, D), suggesting that it was more robust and composed of a tougher material than the outer layer. It is also possible that the inner layer was replaced by clay minerals during early diagenesis. The associated swellings around the outer layer might represent the accumulation of decay fluids (Figs. 6F, 9J). The conical structure connects the stem to the calyx and is always preserved with a similar shape (e.g. Figs. 5A, 11), suggesting it was more rigid and not flexible. The lowest part of the digestive tract is the most reflective part of the animal and appears black with a granular aspect in some specimens. Some specimens show evidence of sediment at the base and near the top of the calyx and just below the external sheath (e.g. Figs. 8B, 12D). This sediment is interpreted as the infilling of small holes in the external sheath probably connecting with the internal part of the calyx.

Among specimens of similar sizes, subtle variations in the shape of the calyx could be interpreted as resulting from a direct consequence of decay (resulting in the collapse of selective tissues) or alternatively from variations in the angles of burial. However, because there are no visible differences in preservation among specimens showing such variations in shape, it is not clear how decay alone could have had an impact and why it would have only affected some but not all specimens of the same size on the same bedding planes. Accordingly, differences in angles of burial among specimens might have played a more important role. Whereas angle of burial could have an influence on the length of the specimens and outline shape, it would be inconsequential for the maximal width, given that the stem and calyx are roughly circular in cross-section. Importantly, few specimens show deformations along the stem (i.e. compression artifacts which would be expected if the stems were buried at an angle) suggesting that most specimens were buried parallel to bedding. For specimens of similar widths and lengths, variations in the shape more likely represent a genuine feature of the animal and demonstrate the extent of flexibility of the stem and calyx encountered in the population (Fig. 10, Table 2).

### Systematic Palaeontology

Unranked stem-group bilaterian

Phylum Uncertain

Family SIPHUSAUCTIDAE fam. nov.

#### Diagnosis

Soft-bodied stalked metazoan with a large ovoid to subrectangular-shaped calyx lodging a prominent hexaradial filter feeding apparatus and a bilayered stem.

Genus SIPHUSAUCTUM gen. nov.

urn:lsid:zoobank.org:act:72FFDBED-21C6-4789-B4F4-34893910A276

*Type species. – Siphusauctum gregarium*, by monotypy.

#### Etymology

Derived from the Latin *siphus*, meaning cup or goblet, and *auctus*, meaning large, referring to the shape and size of the animal.

#### Types

Holotype: ROM 61414 (Fig. 3). Paratypes: ROM 61413 (Fig. 2), ROM 61415 (Fig. 4), ROM 61421 (Fig. 7). *Other material examined.* – 1,133 specimens, all held in the ROM collections.

#### Occurrence

Tulip Beds locality of the lower Middle Cambrian Campsite Cliff Shale Member of the Burgess Shale Formation on Mount Stephen, British Columbia, Canada [13]. One poorly preserved specimen from the Middle Cambrian of Utah (Lieberman, pers. comm., 2009) is tentatively referred as *Siphusauctum* sp., and might represent a different species.

#### Diagnosis

Soft-bodied, gregarious, stalked and upright metazoan, with a chalice shaped body, divided into three distinct parts: a prominent calyx, attached to a narrow stem terminating in a small, bulbous or flat holdfast rarely wider than the stem. Known maximum dimensions, 223 mm in total height and 48 mm in width (at calyx). Calyx, box-shaped, or ovoid, approximately circular in cross-section with rigid conical lower quarter attached to stem. Maximal width at a mid-height narrow ridge, width tapering either sharply or smoothly towards the top. Calyx covered with a flexible and thin external sheath representing the margins of a large filtration chamber. External sheath smooth with the exception of six small openings at base of calyx, an opening for the anus at the top, and indistinct openings around the anus. Internal structures include a central and prominent sac-like gut enclosed by a tube representing the margins of the body cavity. Gut differentiated into three main zones, an ovoid lower tract near the base of the calyx, grading into a bulbous mid-gut, then tapering into a straight and thin upper intestine projecting upwards to a central terminal anus. The gut is enclosed within a body cavity that is surrounded by hexaradial filter-feeding segments arranged longitudinally and filling most of the calyx. The outer surface of each segment consists of fine, diagonally orientated, parallel striae. Internally, a pair of thick transverse comb-like grooves (>30 pairs per segment) occupies most of the width and height of the segments. Grooves taper to a thin point towards the body cavity and connect to a larger groove that extends from the top of the calyx to the level of the lower gut along the outer edge of the segments. Stem roughly equal to or up to three times the length of the calyx, flexible but generally straight with uniform width, divided into an inner and an outer layer.

#### Discussion

*Siphusauctum* has sufficient unique morphological characteristics to erect a new genus within a new family, see discussion below.

SIPHUSAUCTUM GREGARIUM sp. nov.

urn:lsid:zoobank.org:act:9FBD8D62-BEDF-465A-986F-5765A05F93CA

Figures 2, 3, 4, 5, 6, 7, 8, 9, 10, 11, 12, 13

#### Etymology

Derived from the Latin *gregalis*, meaning flock or part of a herd, referring to the gregarious nature of this animal.

#### Diagnosis

As for the genus.

Description

#### General shape and dimensions

This soft-bodied animal has a chalice shaped body (e.g. Figs. 2, 14) with a small rounded or flat holdfast at the base, a narrow stem, and a large bulbous calyx. Specimens range from 19 mm to 223 mm in length and 6.6 mm to 49.8 mm in width, with the stem representing roughly half this dimension, and the mean width at the calyx is 29.6 mm (Table 2). The largest animal is 223 mm long including the calyx and stem (although the complete length of the stem is unknown) (Fig. 5A). The general size variations of the animal and the ratios of correlated measurements are summarized in Table 2.

#### Stem

The stem is a long narrow structure that extends from the base of the calyx to the top of a small holdfast (e.g. Fig. 2, Table 2). The stem ranges in length from 9.5 mm to 147 mm, representing one to three times the length of the calyx. Its width is generally constant along its length. The wide range of lengths recorded suggests the stem was able to expand and retract. Some specimens may be slightly curved, suggesting flexibility (e.g. Fig. 9E, F) but overall, the stem appears to be composed of a relatively recalcitrant material (see also taphonomy section). The stem is clearly divided into two parts referred here as the inner and outer layers. The inner layer originates from within the base of the calyx (see below, stem and calyx attachment), varies from 0.7 mm to 3.7 mm in width and represents about a third of the total stem diameter (e.g. Fig. 9). The inner layer is usually of consistent width along its entire length until it intergrades into the holdfast, where it may increase to twice its width. The outer layer also originates from the base of the calyx and mantles the entire inner layer until the top of the holdfast. At this point it either stops abruptly or tapers gently just short of the holdfast (e.g. Fig. 9). The surface of the outer layer is usually smooth but sometimes shows evidence of small undulations or wrinkles (e.g. Fig. 9A, H), confirming the flexible nature of this organ

#### Holdfast

The holdfast is preserved in the same manner as the inner layer of the stem and appears to be its continuation. Holdfast width ranges from 1.8 mm to 10.8 mm, which is generally twice to three times the width of the inner layer and is rarely wider than the maximal diameter of the stem. The shape of the holdfast ranges from a round disc with a flat base, to bulbous or globular (Fig. 9). While some of this variation could be due to the angle of burial, the globular holdfasts tend to be covered more fully by the outer layer of the stem as compared to the discoidal holdfasts; the latter tend to have the outer layer of the stem tapering abruptly above it. The shape of the holdfast itself does not seem to be correlated with size of the animal. Discoidal and globular holdfasts are found in both small and large specimens. The difference in holdfast morphology is therefore considered biologically significant, and may be the result of the animal actively controlling the shape of its holdfast.

#### Stem and calyx attachment

The base of the calyx is characterized by a conical structure with smooth margins. This is rarely deformed and always occurs in even the most decayed specimens, suggesting that the calyx was rigid in life (e.g. Figs. 5, 11). Distal to the calyx, the outer margin of the conical structure merges with the outer layer of the stem. Differences in preservation, as noted above, between the two structures suggest however that they were functionally separate. The inner layer of the stem extends into the base of the conical structure and also has a conical shape in the lower part of the calyx (Fig. 11A, E, F). This structure is referred to here as the inner cone. The inner cone follows the shape of the external conical structure, and the lowermost gut is generally situated just above the top of this structure. At least one specimen (Fig. 5C, D, ROM 61417) shows a tubular element within the inner cone continuing through the inner layer of the stem, indicating that there may be internal canalization that is not normally preserved. The length of the conical structure (defined as the distance from the top of the stem to the base of the stomach) varies from 1.8 mm to 10.8 mm.

#### Size variations and overall shape of the calyx

The calyx varies in size, with a length of 9 mm to 76.4 mm and a width of 6.6 mm to 49.8 mm (see Table 2). Complete calyxes that show no sign of pre-burial decay are generally widest at the midline, but they can vary in width at the top of the calyx. Some calyxes retain a roughly similar width along their length with the widest part at the midline, others narrow upwards to form an oval shape (Fig. 12). While angle of burial might have an effect on shape of the calyxes, most of the variations observed are interpreted as evidence that the external sheath was flexible and deformable with the ability to contract and expand. As in the holdfast, there is no evidence of an allometric control of the shape of the calyx.

#### External sheath

A thin external sheath covers most of the calyx. Where the calyx is at its widest, a gathering of the external sheath forms a small protrusion on the side of the calyx (e.g. Figs. 10, 12). The position of this protrusion is roughly consistent across specimens regardless of their size. At the base of the calyx, the external sheath tends to fold on either side of the conical structure (e.g. Fig. 11 A, B, C). The external sheath is perforated by the anus, possible indistinct openings circle the top of the calyx, and six openings pierce the base of the sheath between the internal comb-like segments (Fig. 8 and see below). The variation in shape of the internal elements at the top of the calyx gives a crenulated aspect to the external sheath (e.g. Figs. 10, 12) and suggests the presence of openings. However, preservation is too poor to determine their nature, shape and numbers.

#### Comb-like segments

The bulk of the calyx comprises the hexaradial comb-like segments, orientated with their long axis along the length of the calyx. Fine suture lines are located along the long axis of the comb-like segments in some specimens and appear to be the site of their attachment to the external sheath (Fig. 3). The six segments merge near the top of the conical structure and extend up to the top of the calyx (e.g. Fig. 12). Each segment has fine striae along its outer margins, orientated diagonally to the sides of the calyx (e.g. Figs. 3, 4, 13). Internally, the segments consist of a centrally located longitudinal groove, and roughly orthogonal to it are two symmetrical rows of about 30 regularly spaced transverse grooves (e.g. Fig. 3). The rows of transverse grooves generally widen towards the longitudinal grove and are thicker and longer near the base of the calyx, and narrower and shorter towards the top of the calyx (Figs. 2, 3, 4). Although the comb-like segments do not show variation in shape, they do in terms of position. It appears their configuration, either tightly packed together or expanded, accounts for observed variation in calyx width. This difference is most noticeable at the top of the calyx where, if the segments are expanded, the calyx has a corona-like appearance (Fig. 12C, F).

#### Gut

The gut is composed of a tripartite digestive tract with a terminal anus arranged along the longitudinal axis of the calyx (e.g. Figs. 13, 14). A dark and very reflective sub-circular mass, positioned just above the conical structure at the base of the calyx, is interpreted to be the lowest part of the digestive system, possibly representing the stomach (Fig. 13). The lower digestive tract is the widest part of the gut and ranges from 3 mm to 15.9 mm in width and 1.7 mm to 9.2 mm in length. This area narrows upwards and grades into a bulbous area. This is interpreted as the middle part of the digestive tract, and in turn it tapers along its length to form a narrow tube. This represents the upper digestive tract or intestine, terminating in a central anus (Fig. 13) near the top of the calyx. The gut is located within a central body cavity, as evidenced by the occurrence of a tube around the digestive tract. The precise position of the mouth is unknown but was presumably located around the area between the base of the comb-like segments and the stomach.

## Discussion

### Mode of life

The unusual morphology of this animal and the lack of extinct or extant analogues results in difficulties when establishing its mode of life. Despite these difficulties there are enough morphological clues to speculate on how it lived. The large size, stemmed nature, overall shape, and internal characters imply it was an active and semi-sessile benthic filter-feeder. The radially arranged comb-like elements are interpreted to function as a filter feeding structure. We suggest that expansion and contraction of the comb-like elements, and possibly the external sheath, created a pumping mechanism which allowed active water circulation through the calyx. Expansion of the calyx would result in water being drawn through the openings at the base and contraction would have resulted in the expulsion through openings at the top. It is possible that water might have exited through the openings at the base of the calyx as well. The fine striations at the surface of the comb-like elements could represent remnants of connective tissues belonging to muscle bands or less likely could be part of a filtration structure. Similar fine striae are present in the possible stem-group cephalopod *Nectocaris* (fig. 1d and suppl. fig. 12 in [5]) with a similar distance 0.1–0.15 mm between each striation. While *Nectocaris* is unrelated to *Siphusauctum*, these striae point to analogous structures.

Active pumping would require a network of circular muscles in the calyx of *Siphusauctum* and, although muscles themselves are not preserved in the Burgess Shale (e.g. [18]), it is conceivable that connective collagenous tissues which are less prone to decay could be preserved in both organisms. Presumably, the food would have passed from the transverse grooves to the six central grooves belonging to each of the comb-like elements, via the beating of putative cilia (not preserved). Food particles would have circulated down towards the gut, through a central mouth which has not been identified, but is suggested by the concentration of organic matter in this area. The digested food could have been expelled through the terminal anus and transported away without contaminating the inhalant flow of water.

Although the Ctenophora are not considered to be closely related to *S. gregarium* (see below), the apparent similarity between the comb rows of ctenophores and the comb-like segments of *S. gregarium* warrants functional comparison. Modern ctenophores have eight rows of equally spaced combs (plates of hundreds of fused cilia) found externally on the body that function in locomotion when beaten together synchronously. Primitive ctenophores have been recognized in both the Burgess Shale and Chengjiang faunas and differ from modern species by having up to 24 comb rows in some of the Burgess Shale forms and lacking tentacles [19]. The narrow and abundant comb rows of the Burgess Shale ctenophores are not morphologically comparable to *S. gregarium*. However, the putative Chengjiang ctenophore, *Trigoides aclis*, has four to eight broad comb rows [20] that are more similar to the comb-like segments in *S. gregarium*. Despite some similarity, the comb-like segments are not considered morphologically equivalent, because *S. gregarium* has segments that vary in both width and thickness and extend from either side of a longitudinal groove within the calyx. This type of variation is not seen in fossil or extant ctenophores. The presence of these structures internally rather than externally also suggests that they are not functionally comparable, and a locomotory function is not considered for the comb-like elements of *S. gregarium*.

Although *S. gregarium* lived in large groups, there is no evidence of any organic connection between individual specimens, suggesting they were not colonial (i.e. each specimen is a solitary individual). There is no indication of bulging in the stem or calyx which could indicate a particular type of propagation. The presence of a single specimen possibly comparable to *S. gregarium* in the Middle Cambrian of Utah (Lieberman, pers. comm. 2009), would suggest that members of this group occurred at least along the current Western Laurentian sea board during the Middle Cambrian.

**Figure 17:**
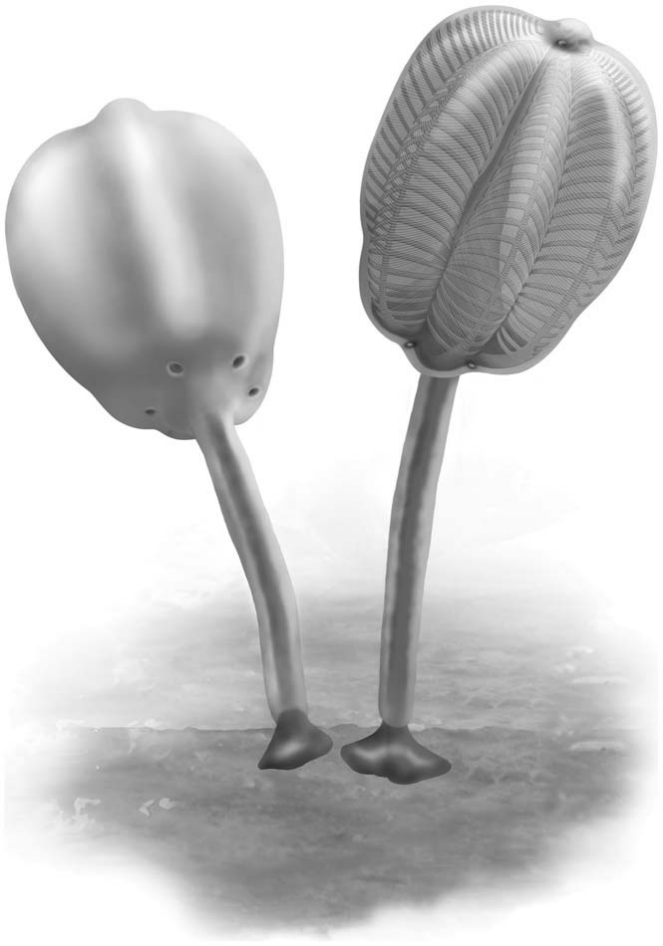
Reconstructions of *Siphusauctum gregarium* n. gen. and n. sp. Left, outer appearance. Right, transparent view through the external sheath to show the internal anatomy of the calyx. (© Marianne Collins).

*Siphusauctum gregarium* was probably well adapted to Proterozoic-style soft substrates [21]. The presence of fragile hexactinellid sponges like *Diagoniella* and large sponges [22] in the Tulip Beds, supports low sedimentation rate and the idea that the environment was relatively deep and not under the influence of strong currents. Also, given the presence of soft-sediment dwellers (e.g. priapulids), it is unlikely that the seafloor was very firm, and would not have provided a compact base for *S. gregarium* to attach. The lack of a large holdfast and variation in holdfast morphology suggest that *S. gregarium* lived in a very low current environment and/or was not permanently anchored. The holdfast would have been located within the flocculent upper mud layer, with the position of the outer layer on the stem giving the approximate level of the water-sediment interface (Fig. 17). We interpret the holdfast acting as a temporary anchor. It is possible that the holdfast could retract partially within the stem, as suggested by the presence of bulbous holdfasts with an extended outer layer. Such variation could have led the holdfast to detach itself from the substrate. Because the length of the stem varies greatly, it suggests that the inner layer of the stem might have been filled with a hydrostatic fluid; presumably allowing the holdfast to expand and contract.

As a facultative sessile, stalked filter feeder, the ecological strategy of *S. gregarium* has no direct counterparts with any other organisms, with the exception perhaps, of *Dinomischus* [23] which is also interpreted as a shallow sediment sticker [21], albeit with important differences (see below). Comparisons with other shallow sediment stickers, in particular from the Ediacaran, are tenuous. Among the forms within the rangeomorphs which have a holdfast and a stem, the structure of the frond differs fundamentally in being flat and elongate and having a fractal geometry (e.g. [24]). *S. gregarium* belongs to the highest tier (10 to 50 cm) in the Burgess Shale community and joins a few monaxonid sponges, *Leptomitus* and *Wapkia* which also occur in the Tulip Beds [22], and *Mackenzia*, a putative sessile cnidarian [25]. A marked difference between *S. gregarium* and the aforementioned species is that *S. gregarium* is abundant and certainly represented a significant biomass in its habitat, which contrasts with other Burgess Shale localities, such as the Walcott Quarry, where most of the sessile filter feeders occupy a low to middle tier [2].

### Biological affinities

Among extinct and extant bilaterians, *S. gregarium* superficially resembles organisms from a range of phyla, embracing entoprocts to tunicates. However, the absence of convincing homologies with any of these phyla suggests no close affinities with *S. gregarium*.

**Figure 18:**
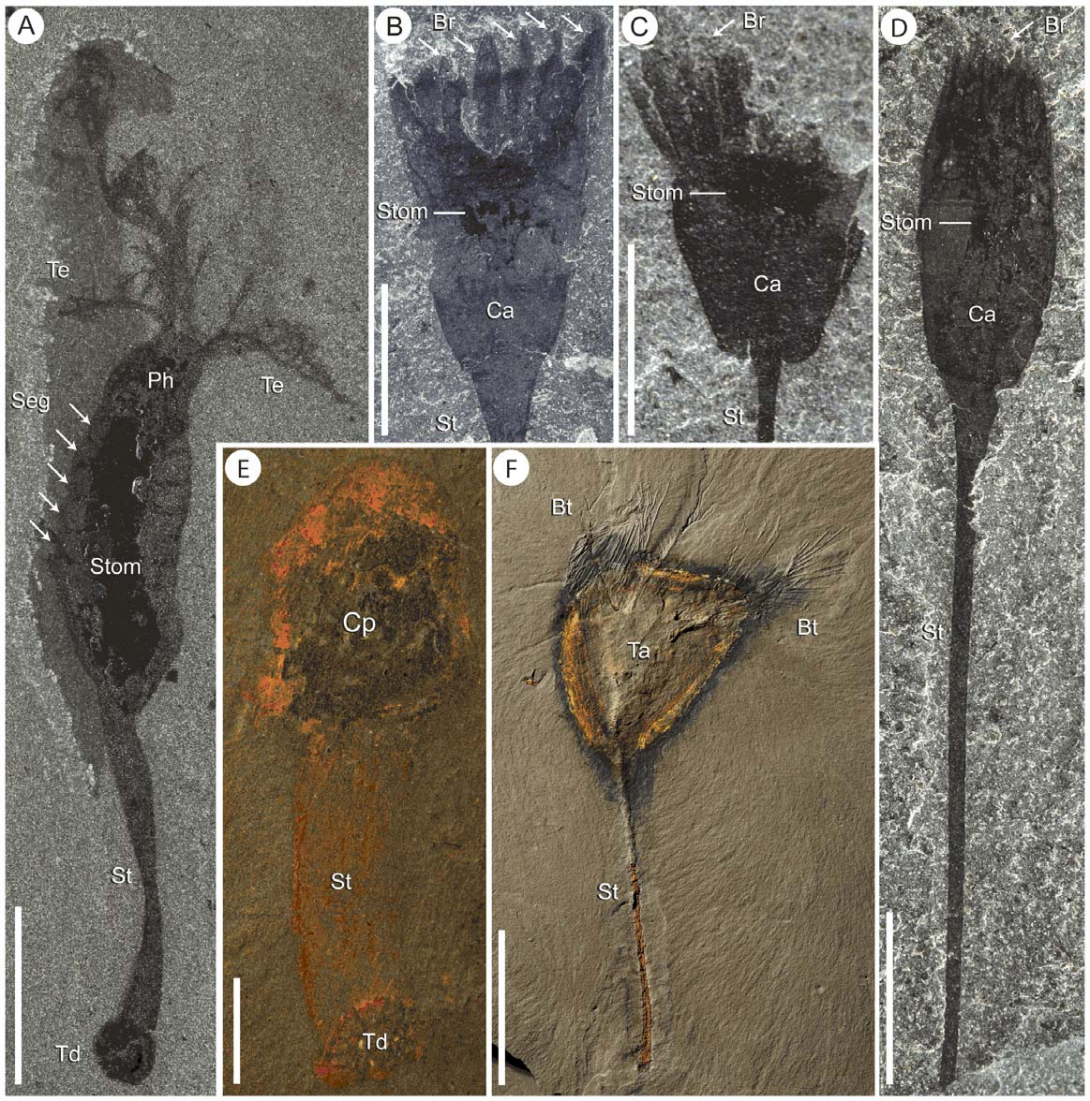
Other stalked animals from the Burgess Shale. A, *Herpetogaster collinsi*, primitive ambulacrarian (ROM 58037). B–D, *Dinomischus isolatus*, problematica. B, holotype (USNM 198735), C, paratype, showing a flatter base of the calyx (MCZ 1083), D, paratype showing long narrow stem and bracts pulled together at the top of the calyx (ROM 32573). E, *Priscansermarinus barnetti*, an enigmatic stalked animal with a thick stem (ROM 36064). F, *Lyracystis radiata*, a basal echinoderm with a long stem, cup shaped calyx (here surrounded by a halo of pyrite) and branchlets (ROM 57229). Scale bars: A–E = 5 mm, F = 30 mm. Abbreviations: Br - Bract, Bt - Branchlet, Ca - Calyx, Cp - Chitinous plate, Ph - Pharynx, Seg - Segment, St - Stem, Stom - Stomach, Ta - theca, Te - Tentacle, Td - Terminal disc.

Among the non-bilaterians, the oval shaped Burgess Shale ctenophore, *Ctenorhabdotus* [19], has a gross morphology that is similar to the calyx of *S. gregarium*. However, as discussed in the previous section, the ctenophore comb rows are not comparable. Therefore, in addition to lacking a stem with holdfast and a defined gut within a body cavity, these animals are not considered to have any close biological affinity with *S. gregarium*. A similar conclusion is reached with the enigmatic attached leaf-shaped animal *Thaumaptilon* [25] from the Burgess Shale. This animal may have affinities with some frondose Ediacaran forms [25] or represent a potential stem group cnidarian [26], but affinities to *S. gregarium* seem very remote. *Priscansermarinus* [27], another problematic stalked Burgess Shale animal originally thought to be a barnacle, is most likely unrelated to this group [28]. This animal has a small holdfast structure and a short deformable stolon. Like *S. gregarium*, this animal was gregarious, had a central visceral mass in the calyx and both animals lacked external tentacular elements. Although the calyx region appears incomparable with *S. gregarium* (Fig. 18E), a revision of *Priscansermarinus* is needed before more precise conclusions about the affinities of these two organisms can be formulated.

Within the deuterostomes, some crinoids have a bulbous calyx on a long stem; however, they have arms with pinnules attached to the aboral region and possess a distinctive stereom. Stem group echinoderms are also found in the Burgess Shale; e.g. *Lyracystis*, a tall suspension feeding echinoderm with three V-shaped arms [29], but a close affinity is not recognized (Fig. 18F). Tunicates have a sessile adult form, but their internal anatomy is incomparable with *S. gregarium*. There is no evidence of a pharynx, incurrent and excurrent siphons or oral tentacles in *S. gregarium*, and tunicates do not have any feeding structures that are similar to the striated elements in the comb-like segments. *Phlogites*, a chalice shaped animal with a stalk, cup-shaped calyx and five branched tentacles, has been compared with the lophophorates [30], entoprocts and the gnathiferans [31], but its position is probably closer to ambulacrarians [32]. *Phlogites* possesses branched tentacles and an anus on the side of the calyx [31], features that are not known in *S. gregarium*. Despite a similar shape of the stem and calyx, both animals are probably unrelated. *Herpetogaster*, another tentaculate animal from the Burgess Shale related to *Phlogites* [32], possesses a disc at the end of a flexible stolon which could have presumably functioned like a holdfast (Fig. 18A). The flexible stolon had an internal tube, potentially originally filled with some hydrostatic fluids which could have functioned for controlling the length of the stolon. Such feature is vaguely reminiscent of the inner layer of the stem in *S. gregarium*. However, like *Phlogites*, it is more likely that adaptation to a semi-sessile lifestyle for a bilaterian filter-feeder organism encompasses similar transformations, from the development of a similar anchoring mechanism, and presence of a flexible stolon, to the development of a cone shaped calyx. All these features are most likely convergent in various groups.

*Dinomischus isolatus*, another stalked animal from the Cambrian, whose biological affinities have yet to be conclusively resolved, may represent the closest relative to *S. gregarium. D. isolatus* is a solitary stalked filter feeder with a calyx sitting on a long stalk and surrounded by a ring of bracts [23] (Fig. 18B, C, D). A second species of *Dinomischus* (*D. venustus*) was described by Chen et al. [33] from the Chengjiang lagerstätte and an undescribed species has been reported from the Kaili biota [34]. *Dinomischus venustus* is morphologically similar to *D. isolatus*, but was described with a long rod-like extension from the top of the calyx. This feature was considered to represent a long anal pore [33]; however, Chen and Erdtmann [35] disagreed on the interpretation of this structure. Erdtmann argued that the structure is the stem, twisted under the calyx, especially because the extension is similarly preserved, and has the same thickness as, the stem. Chen argued for the anal pore interpretation, because the structure appears to be on the same plane as the calyx [35]. The alternative interpretation, with the stem folded on top of the calyx, is probably more parsimonious, especially because sediment separates the two structures, and the base of the stem is not visible on the fossils. *Dinomischus* shares with *S. gregarium* a long, narrow, stalk and a large stomach sac near the base of the calyx, which preserves as a mass of highly reflective black carbonaceous minerals. The stem in *Dinomischus* does not seem to be organized with an inner and outer layer. However, this could be taphonomic, with the outer layer not preserved, as can be seen in a number of *S. gregarium* specimens. Both organisms have a distinctive rigid conical structure that gives shape to the base of their calyx and acts as the base from which the other elements attach to. A ring of filter feeding elements encircles the calyx and encloses a putative mouth and anus in both animals. The bracts of *Dinomischus* appear to be short and stiff, whereas the segments of *S. gregarium* are much larger, with striated sheaths and feeding grooves. There is at least some indication of wrinkled internal structure in the bracts of *Dinomischus* that may be reminiscent of the striated elements of *S. gregarium* (fig. 5 in Chen et al. [33]). However, to date, these structures are poorly described, and it is unclear if they are comparable. It is also noteworthy that finer details of the bracts could have also been lost, especially since smaller features in small animals like *Dinomischus* are less likely to be preserved. Perhaps the most important differences between the two organisms are the number of the filter feeding elements, with six in *S. gregarium* and about 18 to 20 in *Dinomischus*, and the apparent lack of an external sheath enclosing the bracts of *Dinomischus*. Overall the similarities suggest the two organisms may be related. However, recognizing conclusive homologous characters between these two organisms may be difficult, and detailed studies of the available material of *Dinomischus* would be required to confirm or dismiss this hypothesis.

Amongst extant protostomes, comparisons with the entoprocts and ectoprocts are equally difficult to support. While previously it has been suggested that extant entoprocts are possibly miniaturized descendents of a *Dinomischus*-like animal [23], [33], it is difficult to make any comparisons with *S. gregarium*. Entoprocts and ectoprocts are two phyla of tiny stalked, sessile, solitary or colonial animals, but the morphology of their calyx is not comparable, and the affinities of these two groups remains contentious (see discussion in Caron et al. [32]).

Regardless of whether *S. gregarium* and *Dinomischus* are related, possession of a gut would suggest a grade of organization above sponges, cnidarians and ctenophores, possibly as a stem-bilaterian or within bilaterians. However, if radial organisms are in fact derived from a bilaterian ancestor [36], the presence of a gut itself might not be particularly informative. Like other fossil taxa [4], *S. gregarium* may elude the title Problematica, by collection of more specimens (potentially from various sites) with better morphological details preserved to resolve preservational artifacts and examination of the similarities with other animal groups rather than focusing on the differences. In the case of *S. gregarium* the problems of its biological affinity are not due to poor preservation or lack of specimens but rather the lack of phylogenetically informative characters, similar to the difficulties in resolving the phylogeny of the entoprocts. The lack of informative characters and the potential for so many sister group hypotheses for *S. gregarium* are probably due to its mode of life and the biological constrictions it imposes. The interpretations of mode of life made herein are hypotheses based upon the reconstruction of a large collection of animals and considerations for potential taphonomic variations as well as comparisons to extant taxa, however the very unusual arrangement within the calyx limit these comparisons. Discoveries of new specimens and species in other Burgess Shale-type deposits, in particular from the Chengjiang biota, and a detailed comparison with *Dinomischus* might help resolve the affinity of this animal in the future.
